# Enthalpy–Entropy Compensation in the Promiscuous Interaction of an Intrinsically Disordered Protein with Homologous Protein Partners

**DOI:** 10.3390/biom11081204

**Published:** 2021-08-13

**Authors:** Jaka Kragelj, Thibault Orand, Elise Delaforge, Laura Tengo, Martin Blackledge, Andrés Palencia, Malene Ringkjøbing Jensen

**Affiliations:** 1 Université Grenoble Alpes, CNRS, CEA, Institut de Biologie Structurale, Grenoble, France; Jaka.Kragelj@UTSouthwestern.edu (J.K.); thibault.orand@ibs.fr (T.O.); elise.delaforge@gmail.com (E.D.); ltengo@embl.fr (L.T.); martin.blackledge@ibs.fr (M.B.); 2 Institute for Advanced Biosciences, Structural Biology of Novel Targets in Human Diseases, INSERM U1209, CNRS UMR5309, Université Grenoble Alpes, Grenoble, France; andres.palencia@inserm.fr

**Keywords:** NMR spectroscopy, isothermal titration calorimetry, intrinsically disordered protein (IDP), mitogen-activated protein kinase (MAPK), enthalpy–entropy compensation, folding-upon-binding, chemical exchange saturation transfer (CEST)

## Abstract

Intrinsically disordered proteins (IDPs) can engage in promiscuous interactions with their protein targets; however, it is not clear how this feature is encoded in the primary sequence of the IDPs and to what extent the surface properties and the shape of the binding cavity dictate the binding mode and the final bound conformation. Here we show, using a combination of nuclear magnetic resonance (NMR) spectroscopy and isothermal titration calorimetry (ITC), that the promiscuous interaction of the intrinsically disordered regulatory domain of the mitogen-activated protein kinase kinase MKK4 with p38α and JNK1 is facilitated by folding-upon-binding into two different conformations, despite the high sequence conservation and structural homology between p38α and JNK1. Our results support a model whereby the specific surface properties of JNK1 and p38α dictate the bound conformation of MKK4 and that enthalpy–entropy compensation plays a major role in maintaining comparable binding affinities for MKK4 towards the two kinases.

## 1. Introduction

Over the last two decades, it has become increasingly clear that a large fraction (up to 40%) of the human proteome is intrinsically disordered or contains disordered regions of significant length [1,2,3,4]. These intrinsically disordered proteins (IDPs) play important regulatory roles in many biological processes, and they have been intimately linked to a number of human diseases underlining the importance of understanding their conformational properties and interactions at the molecular level. IDPs rely on short sequence segments (linear motifs) to mediate interactions with partner proteins [5,6], and several different binding modes have been identified including the folding-upon-binding mechanism, where the IDP folds into a specific conformation on the surface of the partner protein [7,8,9,10,11,12,13]. It has been suggested that folding-upon-binding results in an overall unfavorable entropic contribution to binding thereby allowing IDPs to achieve specific binding without displaying a concomitantly high binding affinity [14,15]. This would have advantages, for example, in signaling processes where reversible interactions are preferred [16]. However, in some cases, the large unfavorable entropic contribution may be partially or entirely compensated by a large favorable enthalpic contribution upon binding (enthalpy–entropy compensation) [17,18,19], by the pre-formation of secondary structure in the free-state ensemble of the IDP [20,21], and/or by a gain in solvent entropy from the release of water molecules at the binding interface that is usually of hydrophobic character [22,23]. For these reasons, the link between binding specificity and affinity is not straightforward and has to be independently evaluated for each IDP complex.

In addition to these thermodynamic considerations, IDPs can be promiscuous in nature i.e., they can interact with different partner proteins, even via the same linear motif [24]. One prominent example is the C-terminal intrinsically disordered domain of the tumor suppressor p53 that folds into different conformations (α-helix, β-sheet or random coil) upon binding to different partners [25]. It is not clear how IDPs achieve this folding into different conformations and, in particular, how this feature is encoded in the primary sequence of the IDP and to what extent the surface properties and the shape of the binding cavity dictate the final bound conformation. To shed light on this, a more direct comparison of promiscuous IDP complexes in terms of structure, interaction kinetics and thermodynamics is necessary.

We study the promiscuous interaction of an IDP within the mitogen-activated protein kinase (MAPK) cell signaling pathways. The MAPK pathways are essential components of eukaryotic signal transduction networks that direct appropriate responses to cellular stress [26], and four major MAPK pathways have been identified in mammalian organisms: the two ERK (extracellular-signal-regulated kinase) pathways, p38 and JNK (c-Jun N-terminal kinase) [27]. Docking site motifs play crucial roles in mediating signaling specificity in these pathways [28,29]. They are composed of two to three basic residues (arginine or lysine) and three hydrophobic residues (typically valine, leucine, isoleucine, proline, methionine or phenylalanine) according to the following consensus sequence: K/R_1–5_–X_1–6_–Φ_L_–X_1–3_–Φ_A_–X–Φ_B_ (where X is any amino acid type, and Φ indicates a hydrophobic residue) [30]. Docking site motifs are embedded within intrinsically disordered regions of MAPK activators (MKKs), deactivators (phosphatases), substrates and scaffold proteins and bind selectively to one of the three MAPKs (JNK, p38 or ERK) to ensure high fidelity in signal transduction. Thus, for example, all seven human MKKs have N-terminal intrinsically disordered regulatory domains containing docking site motifs that selectively recruit JNK, p38 or ERK leading to specific activation (phosphorylation) of the MAPK by the MKK. One exception is MKK4 that contains an 86-amino acid disordered domain with a docking site motif that can recruit both the JNK and p38 kinases. This makes MKK4 the only MKK that activates two different MAPK pathways [31,32]. This naturally poses the question of how the docking site motif of MKK4 has evolved to become promiscuous in nature, while other docking site motifs show clear selectivity between the p38 and JNK pathways [33].

Here we show, using a combination of nuclear magnetic resonance (NMR) spectroscopy and isothermal titration calorimetry (ITC), that the promiscuous interaction of the disordered regulatory domain of MKK4 with p38α and JNK1 is facilitated by folding-upon-binding into two different conformations, despite the high sequence conservation and structural homology between the two kinases. Our results support a model whereby the specific surface properties of the two kinases dictate the bound conformation of MKK4 and that enthalpy–entropy compensation plays a major role in maintaining comparable binding affinities for MKK4 towards p38α and JNK1.

## 2. Materials and Methods

### 2.1. Sample Preparation

The regulatory domain of MKK4 (residues 12–86 of human MKK4, Uniprot P45985, hereafter named MKK4), the JNK1 kinase (residues 1–364 of human JNK1 (isoform α1) with a C-terminal 6xHis tag, Uniprot P45983) and the p38α kinase (residues 1–360 of human p38α, Uniprot Q16539) were expressed and purified as described previously [34,35]. The regulatory domain of MKK4 was either ^15^N-labeled (for NMR) or unlabeled (for ITC). 

### 2.2. ITC Experiments

ITC measurements of the JNK1:MKK4 complex were performed on a MicroCal iTC200 (MicroCal, Northampton, MA, USA) at 20 °C. Injections of 1.5 μL were carried out every 180 s, 26 in total at a stirring speed of 800 rpm. Prior to the experiments, any impurities or aggregates of MKK4 and JNK1 were removed by size-exclusion chromatography in the ITC buffer (50 mM HEPES pH 8.2, 150 mM NaCl, 10% glycerol (*v/v*), 0.5 mM TCEP). MKK4 with a concentration of 570 μM was titrated into a solution of JNK1 with a concentration of 42 μM. ITC experiments were carried out in the presence of the N-terminal thioredoxin and 6xHis tag on MKK4 to allow for accurate protein concentration determination by UV absorbance using the theoretical molar extinction coefficient calculated from the primary sequence of the fusion protein. A control experiment shows that JNK1 does not bind to the his-tagged thioredoxin [34]. The ITC experiments of the p38α:MKK4 complex were carried out previously [35].

### 2.3. NMR Titrations

All NMR experiments were carried out using ^15^N-labeled samples of MKK4 (residues 12–86) in 50 mM HEPES pH 7.1, 150 mM NaCl, 2 mM dithiothreitol (DTT), 5% (*v/v*) glycerol at 5 °C. The spectral assignments of MKK4 were obtained previously at 5 °C [35]. A titration of ^15^N-labeled MKK4 with unlabeled JNK1 was carried out for the following concentrations of MKK4 and JNK1: 120:0 μM (0%), 120:24 μM (20%), 120:48 μM (40%), 68:46 μM (68%), 50:45 μM (90%). For each titration point, an ^1^H-^15^N HSQC spectrum was measured at a ^1^H frequency of 600 MHz, and the intensities were extracted and normalized to the intensities in the spectrum recorded in the absence of JNK1 by accounting for the difference in protein concentration and the number of scans. 

^15^N spin relaxation rates *R*_1_ and *R*_1__ρ_ in MKK4 were measured at 5 °C and a ^1^H frequency of 600 MHz using HSQC-detected pulse sequences [36]. A spin lock field of 1.5 kHz was used in the *R*_1__ρ_ experiments. The decay of magnetization was sampled at 70, 130, 10, 90, 230, 30, 210, 50, 170, 1 ms with a repeat at 70 ms for the *R*_1__ρ_ experiments, and at 0, 0.6, 0.08, 1.6, 0.4, 1.8, 1.04, 0.8, 0.2 s with a repeat at 0.6 s for the *R*_1_ experiments. Errors on the relaxation rates were estimated from Monte-Carlo simulations using the noise in each plane of the pseudo-3D spectra as errors on measured peak intensities. The concentration of MKK4 (200 μM) was kept constant, while varying the amount JNK1 kinase: 0%, 10%, 30% and 50% molar fraction for the *R*_1__ρ_ experiments and 0%, 30% and 50% for the *R*_1_ experiments. The measured ^15^N *R*_1_ rates are identical within experimental error for all molar fractions of JNK1, allowing us to use the most precise *R*_1_ dataset (molar fraction of 0% JNK1) to correct for resonance offset [37]. To this end, we used the following equation: *R*_2_ = (*R*_1__ρ_ − R_1_cos^2^θ)/sin^2^θ where θ = arctan (ω_1_/Δω), ω_1_ being the spin lock field strength (1.5 kHz in this case) and Δω the resonance offset from the ^15^N carrier frequency. Errors on the derived *R*_2_ relaxation rates were calculated from the experimental errors on *R*_1__ρ_ and *R*_1_.

To estimate a lower limit for the transverse relaxation rate, *R*_2_, of the MKK4:JNK1 complex, we first assumed that the rotational correlation time of JNK1 is similar to its structural homologue p38α. A rotational correlation time of τ_c_ = 21.8 ns of p38α at 25 °C was determined previously from a global analysis of ^15^N relaxation rates. Under our experimental conditions (5 °C and 5% glycerol), we obtained a correlation time of 47.1 ns by accounting for the change in viscosity [38] using the Stokes–Einstein equation. Using this value for the correlation time, and by taking into account dipolar and chemical shift anisotropy (CSA) contributions to the relaxation, we estimate an average *R*_2_ rate of 69 s^−1^ at 600 MHz. This value represents a lower value for the relaxation rate in the MKK4:JNK1 complex, as the disordered chain of MKK4 presumably would induce a dragging effect leading to a significantly larger τ_c_ value, as predicted previously [39].

### 2.4. NMR Exchange Experiments

The ^15^N chemical exchange saturation transfer (CEST) experiments [40] were carried out on a sample of 250 μM MKK4 with 0 or 10% molar ratio of JNK1 at a ^1^H frequency of 700 MHz or 850 MHz. A *B*_1_ saturating field of 30 Hz (free MKK4) and 21 Hz (MKK4 with JNK1) was used during a constant period of 300 ms. The data were analyzed according to a two-site exchange model using ChemEx (https://github.com/gbouvignies/ChemEx accessed on 25 May 2021) as described in the main text. 

The NMR exchange experiments (CEST and CPMG relaxation dispersion) on MKK4 with the p38α kinase were previously acquired [35]. Here, we used the dataset of MKK4 (247 μM) with 6% molar ratio of p38α to obtain the chemical shift differences between free and p38α-bound MKK4.

## 3. Results

### 3.1. Interaction Profile of the Regulatory Domain of MKK4 with the JNK1 Kinase

**Figure 1:**
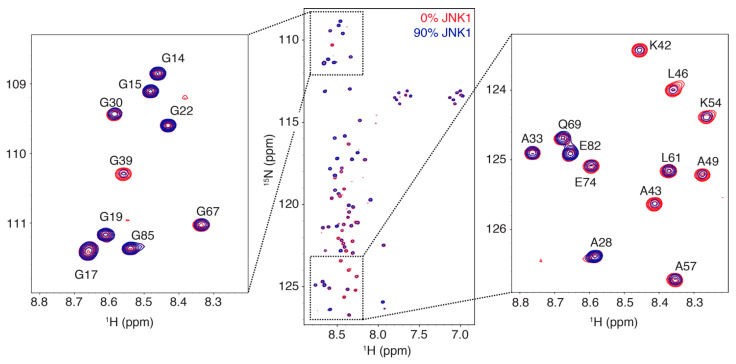
Superposition of the ^1^H-^15^N HSQC spectrum of the intrinsically disordered regulatory domain of MKK4 (residues 12–86) in the absence (red) and presence (blue) of the JNK1 kinase (MKK4:JNK1 molar ratio of 0.9). Two selected regions are shown as zooms with labels indicating the resonance assignments.

**Figure 2:**
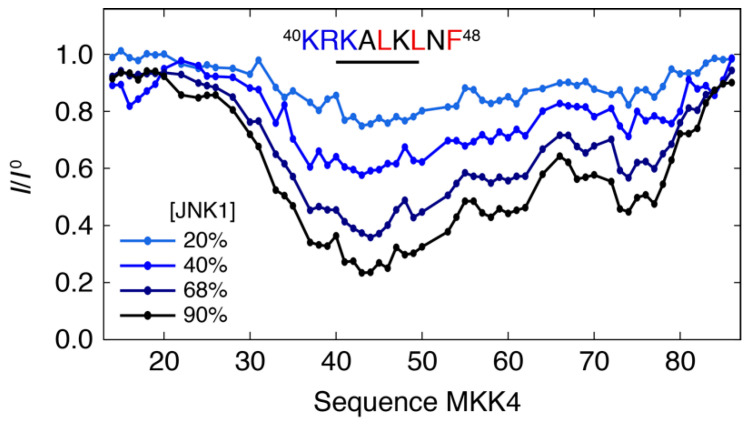
Intensity profile (*I*/*I*^0^) obtained by quantifying the NMR signal intensities in the ^1^H-^15^N HSQC spectrum of the free state of MKK4 (*I*^0^) and of MKK4 with different molar ratios of JNK1 (*I*): 20%, 40%, 68% and 90%. The docking site motif of MKK4 encompasses residues K40-F48 (primary sequence is shown).

We studied the interaction between the regulatory domain of MKK4 (residues 12–86) with the JNK1 kinase by recording ^1^H-^15^N HSQC spectra of MKK4 with increasing amounts of JNK1. Line broadening is observed for multiple residues upon addition of JNK1 demonstrating a clear interaction, while chemical shift changes are negligible (Figure 1). We quantified the intensities of the resonances as a function of the molar ratio of JNK1 showing that the interacting region extends over a large part of the MKK4 sequence with the docking site motif (^40^KRKALKLNF^48^) experiencing the most pronounced line broadening (Figure 2). This suggests that the interaction is mediated by the docking site motif and that the remainder of the chain makes contacts with the surface of JNK1 within the complex. The decrease in intensities within the docking site motif correlates with the amount of added JNK1 demonstrating the absence of significant conformational exchange contributions to the transverse relaxation.

**Figure 3:**
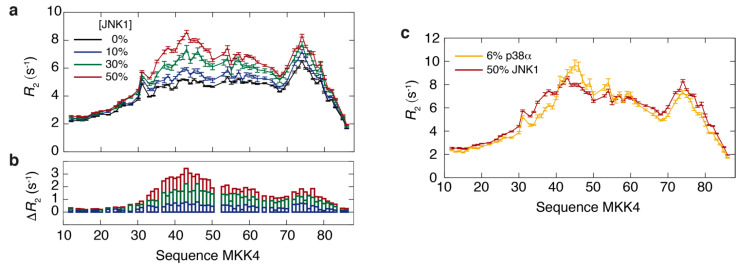
(**a**,**b**) ^15^N *R*_2_ rates in MKK4 measured as function of different molar ratios of JNK1 (0%, 10%, 30% and 50%). Panel (**b**) shows the difference in the rates compared to the free form of MKK4. (**c**) Comparison of ^15^N *R*_2_ rates in MKK4 in the presence of JNK1 (50% molar ratio, red, this work) and p38α (6% molar ratio, yellow, adapted from ref. [35]).

To study the dynamics of the complex in more detail, we acquired ^15^N *R*_1__ρ_ relaxation rates using a spin lock field of 1.5 kHz for different molar fractions of JNK1 (0%, 10%, 30% and 50%). The rates were converted into *R*_2_ relaxation rates by taking off-resonance effects into account (Figure 3a). Only a modest increase in the *R*_2_ relaxation rates for increasing JNK1 concentration is observed (Figure 3b). In the case of fast exchange between the free and the JNK1-bound form of MKK4, the experimental *R*_2_ relaxation rates would be equal to a population-weighted average between the *R*_2_ rate in the free and bound forms. Al-though the *R*_2_ rate of the bound complex is not directly known, we have estimated a lower limit for this rate, $R_{2,\mathrm{bound}}^{\mathrm{lower}}$ = 69 s^−1^, at 600 MHz and 5 °C (see Materials and Methods). The small contribution from this large *R*_2_ rate to the measured relaxation rates in MKK4 suggests that the exchange between free and bound MKK4 is slow on the chemical shift time scale.

This slow exchange behavior is in contrast to the complex of MKK4 with p38α, a paralogue of JNK1 with high sequence and structural similarity (Figures S1–S3), which displays intermediate exchange under the same experimental conditions [35]. The *R*_2_ relaxation rates of MKK4 with 50% and 6% molar ratio of JNK1 and p38α, respectively, are similar, demonstrating the influence of the two different exchange regimes on the mea-sured relaxation rates (Figure 3c). Thus, a much larger contribution from the *R*_2_ rate of the complex is observed in the case of p38α. More generally, the comparison of the relaxation rates shows that the overall interaction profile of MKK4 with the two paralogous MAPKs is remarkably well-conserved, even for residues located outside the docking site motif.

### 3.2. Thermodynamics of the Interaction of MKK4 with the Two MAPK Paralogs

**Figure 4:**
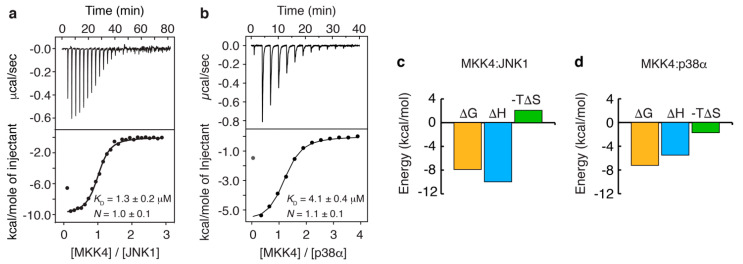
ITC data of the interaction of MKK4 with JNK1 (**a**) and p38α (**b**). Representative data are shown with raw injection heats (upper) and the corresponding specific binding isotherms (lower). The data were analyzed according to a model of *n* identical and independent binding sites. The corresponding dissociation constants (*K*_D_) and stoichiometries (*N*) are reported with error bars corresponding to the fitting error in Origin. The binding free energies were dissected into enthalpic and entropic contributions for both the MKK4:JNK1 (**c**) and MKK4:p38α (**d**) complexes. The ITC data of the p38α complex were previously acquired [35].

Next, we compared the thermodynamic profiles of the MKK4:JNK1 and MKK4:p38α complexes by using ITC (Figure 4). Both complexes show a 1:1 stoichiometry with the JNK1 complex displaying an approximately three times higher binding affinity (*K*_D_ = 1.3 μM) than the p38α complex (*K*_D_ = 4.1 μM). Dissection of the binding free energies into enthalpic and entropic contributions shows that the JNK1 complex is characterized by a small, unfavorable entropic contribution to binding (Figure 4c), while the p38α complex displays a small, but favorable, entropic contribution (Figure 4d). Interestingly, this difference in entropy is compensated by an almost two times higher binding enthalpy for the JNK1 interaction compared to p38α. The observed difference in enthalpy ( ΔΔH = 4.5 kcal/mol) corresponds to the formation of two or three additional hydrogen bonds [41] in the JNK1 complex compared to the p38α complex.

### 3.3. NMR Exchange Experiments of the MKK4:JNK1 and MKK4:p38α Complexes

**Figure 5:**
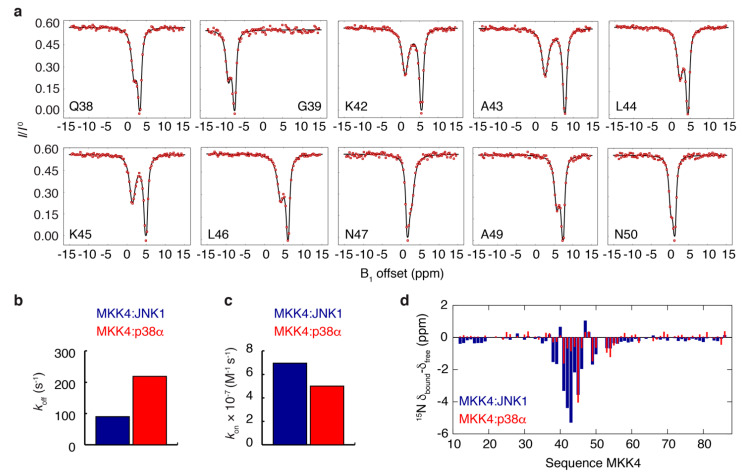
(**a**) ^15^N CEST data of MKK4 in the presence of 10% molar ratio of JNK1. Red circles correspond to experimental data, while full drawn black lines correspond to a simultaneous analysis of the data according to a two-site exchange model. (**b**,**c**) Kinetic constants, *k*_off_ (**b**) and *k*_on_ (**c**), describing the interaction of MKK4 with JNK1 (blue) and p38α (red). (**d**) ^15^N chemical shift differences between the bound and free state of MKK4 derived from analysis of NMR exchange ex-periments (see main text).

To study the origin of the different thermodynamic profiles of the two complexes, we used ^15^N CEST experiments [40] to characterize the MKK4:JNK1 complex. CEST data of the free form of MKK4 show no detectable conformational exchange (Figure S4); however, in the presence of 10% molar ratio of JNK1, the CEST experiments reveal two states in slow conformational exchange corresponding to the free and JNK1-bound form of MKK4 (Figure 5a). In particular, well-separated CEST dips were observed for residues 38–50 containing the docking site motif. Initially, the CEST data were analyzed according to a two-site exchange model for a subset of residues (Q38, G39, K42, A43, L44, K45, L46, N47, A49, N50) showing large chemical shift differences between the free and JNK1-bound form of MKK4 (Figure 5a). The analysis yields an exchange rate constant of *k*_EX_ = 99 ± 5 s^−1^ and a population of the bound state of *p*_bound_ = 8.7 ± 0.1% corresponding to an off-rate of *k*_off_ = 90 s^−1^. Using this off-rate and the dissociation constant determined by ITC, we obtained an on-rate of *k*_on_ = 6.95 × 10^7^ M^−1^s^−1^ for the MKK4:JNK1 complex (Figure 5b).

The same type of analysis was carried out for the MKK4:p38α complex for which ^15^N CEST and CPMG relaxation dispersion experiments were previously acquired using a sample of MKK4 with 6% molar ratio of p38α [35]. Residues showing the largest exchange contributions in the CPMG relaxation dispersion experiments were selected (R41, K45, A49, F53, K54), and a simultaneous analysis of all experimental data according to a two-site exchange model yields *k*_EX_ = 219 ± 6 s^−1^ and *p*_bound_ = 6.2 ± 0.1% corresponding to an off-rate of *k*_off_ = 205 s^−1^ and an on-rate of *k*_on_ = 5.01 × 10^7^ M^−1^s^−1^ for the MKK4:p38α complex (Figure 5b). Our analysis demonstrates that the difference in affinity measured by ITC (Figure 4) for the two complexes mainly reflects a difference in the complex off-rates, rather than the on-rates. 

Next, by fixing the exchange rate constant and the population to the above-determined values in the analysis of the NMR exchange data, we derived the chemical shift differences between the free and bound state for all residues in the MKK4 regulatory domain for the two complexes (Figure 5d). Surprisingly, the chemical shift changes in MKK4 upon binding to the two paralogous MAPKs are very different, with the JNK1 complex showing significantly larger shifts, in particular, for residues R41, K42, A43 and L44 of MKK4.

### 3.4. MKK4 Folds into Two Different Conformations upon Binding to JNK1 and p38α

**Figure 6:**
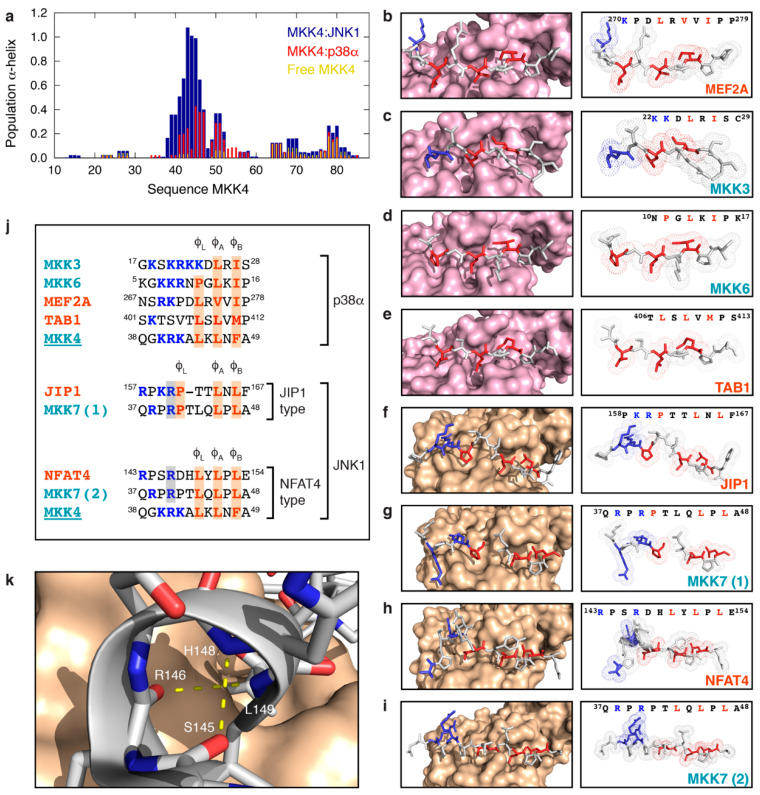
Structural comparison of complexes between docking site motifs and p38α or JNK1. (**a**) Population of α-helix in the free state of MKK4 (yellow) and the MKK4:JNK1 (blue) and MKK4:p38α (red) complexes calculated from the experimental chemical shifts. (**b**–**e**) Structure of p38α (surface representation) in complex with the docking site motifs (sticks) of MEF2A (PDB 1LEW), MKK3 (PDB 1LEZ), MKK6 (PDB 2Y8O) and TAB1 (PDB 4KA3). Labels in turquoise and orange are upstream kinases and scaffold proteins or MAPK substrates, respectively. (**f**–**i**) Structure of JNK1 (surface representation) in complex with the docking site motifs (sticks) of JIP1 (PDB 1UKH), MKK7 conformation 1 (PDB 4UX9), NFAT4 (PDB 2XRW) and MKK7 conformation 2 (PDB 4UX9). (**j**) Structure-based sequence alignment of JNK1 and p38α docking site motifs. Orange shading highlights the residues that occupy the three MAPK hydrophobic pockets, while gray shading indicates positively charged residues that clearly interact with the CD groove as observed in the crystal structures. (**k**) Zoom on the helical turn adopted by the docking site motif of NFAT4 in complex with JNK1. Two hydrogen bonds (residue *i* to *i + 3*) are formed to stabilize the helical structure.

To study the structural changes in MKK4 upon binding to JNK1 and p38α, we used the experimental chemical shifts to calculate the α-helical propensities of MKK4 in the free and JNK1- and p38α-bound states. Although ^15^N chemical shifts are traditionally con-sidered not to be sensitive to the presence of secondary structure [42], advances in accounting for the effect of neighboring residues on ^15^N chemical shifts have rendered them exploitable for secondary structure quantification [43,44,45]. We used POTENCI [46] to obtain the random coil chemical shifts for MKK4 under our experimental conditions (5 °C, pH 7.0), and we calculated the α-helical propensities (averaged over a window of three residues) along the chain of MKK4 using the expected ^15^N chemical shift values for a fully formed α-helix [47] (Figure 6a). The results show that MKK4 folds into two different conformations upon binding to JNK1 and p38α. While MKK4 shows poor helical propensity in complex with p38α, it folds into a helix encompassing residues K40-K45 upon binding to JNK1 (Figure 6a). We note that a conformational selection mechanism can be ruled out in the case of the JNK1 complex, as MKK4 does not show any helical propensity in its free state (Figure 6a) [35]. 

We compared these observations to solved crystal structures of docking site motifs in complex with JNK1 or p38α (Figure 6b–i). We focused on structures of MAPKs in complex with peptides derived from activators (MKKs) [33,34,48], substrates [33,48,49] or scaffold proteins [50]. The structure of p38α in complex with the two activators, MKK3 and MKK6, and the two substrates, MEF2A and TAB1, all show extended conformations of the docking site motifs (Figure 6b–e and Figure S5). A structure-based sequence alignment of the docking site motifs (Figure 6j) reveals that p38α-specific docking sites can accommodate a varying number of residues between the first hydrophobic position (Φ_L_ pocket) and the basic residues (CD groove). This is in agreement with the observation that p38α displays a wider binding groove and a larger distance between the Φ_L_ pocket and the CD groove compared to JNK1 [33].

On the other hand, two types of motifs can be recognized by JNK1 and its narrower docking groove. The docking site motif of the scaffold protein JIP1 binds in an extended conformation (Figure 6f and Figure S5) and is characterized by two residues spanning the Φ_L_ and Φ_A_ pockets with the basic residues immediately preceding the Φ_L_ pocket. In contrast, the docking motif of the substrate NFAT4 has only one residue between each hydrophobic pocket, but with two residues between the basic part of the motif and the Φ_L_ pocket (Figure 6j). To compensate for the narrower binding pocket in JNK1, NFAT4 folds into a one-turn helical element encompassing residues S145-L149 allowing the basic residue R146 to be positioned correctly with respect to the CD groove (Figure 6h,k and Figure S5).

Interestingly, the activator MKK7 shows two different conformations in complex with JNK1 that differ in the specific residues occupying the three hydrophobic pockets (Φ_L_, Φ_A,_ and Φ_B_) [34]. Thus, conformation 1 uses P41, L45 and L47 and conforms to the JIP1-type consensus sequence, while conformation 2 employs L43, L45 and L47 and represents the NFAT4-type consensus sequence [34] (Figure 6g,i,j). In both cases, MKK7 binds in an extended conformation and helical folding is surprisingly not necessary in the case of conformation 2 to adapt to the narrower JNK1 docking groove. The presence of proline residues (P39 and P41) within the MKK7 docking site motif prevents the helical folding that is observed in the case of NFAT4. 

On the basis of our chemical shift analysis and the available crystal structures, we propose that MKK4 binds in an extended conformation to p38α, analogous to other crystallized p38α-specific docking site motifs (Figure 6b–e), and to JNK1 in a helical conformation encompassing residues K40-K45, similarly to NFAT4 (Figure 6h). The fact that MKK4 folds into two different conformations upon binding to the two paralogous MAPKs explains the observed thermodynamic profiles of the two complexes derived from ITC experiments (Figure 4c,d). Thus, the JNK1 complex is characterized by a small, unfavorable entropic contribution to binding due to local helical folding. However, the α-helical folding would result in the formation of two additional hydrogen bonds in the JNK1 complex compared to the p38α complex (Figure 6k) providing a structural explanation for the almost two times higher binding enthalpy for the JNK1 complex compared to the p38α interaction. On the basis of the sequence alignment of the docking site motifs of MKK4 and NFAT4 (Figure 6j), we propose that two intra-molecular hydrogen bonds would be formed between K40CO and A43H^N^ and between R41CO and L44H^N^ of JNK1-bound MKK4. MKK4 binds in an extended conformation to p38α in agreement with the observed favorable entropic contribution and the absence of pre-formed secondary structure in the free state of MKK4 [35].

## 4. Discussion

Despite the importance of docking site motifs in MAPK cell signaling, it has been difficult to establish general rules for their pathway discrimination. A study based on crystal structures and affinity measurements pointed towards an essential role for the intervening region between the consensus positions as the key determinants for specificity [33], while another study based on substrate competition assays suggested that the nature of the hydrophobic residues govern specificity [51]. MKK4 is the only MKK that recognizes two different MAPKs, and we here provide experimental evidence that MKK4 retains its dual specificity by structurally adapting to the different geometry of the binding pockets by folding into two different conformations. This conclusion is in agreement with previous mutational studies based on measurements of binding affinity of MKK4 towards JNK1 and p38α. Thus, the Bardwell group probed the influence of the charged residues ^40^KRK^42^ in the MKK4 docking site motif and showed that R41 is essential for maintaining a high affinity complex with JNK1 [51]. R41 is equivalent to R146 in NFAT4 that directly interacts with the negatively charged CD groove of JNK1, an interaction that is entirely facilitated by the local helical folding. In addition, the Reményi group showed, using fluorescence polarization-based measurements, that the mutation L44P in MKK4 completely abolishes binding to JNK1, while maintaining micromolar affinity towards p38α [33]. This is in agreement with the motif losing its ability to undergo α-helical folding due to the presence of the proline, while retaining its capacity to interact in the extended p38α-compatible binding mode. Thus, these previous mutational analyses, in combination with our experimental chemical shift data, provide strong support for a dual binding mode of MKK4.

More generally, MKK4 is a new example of a promiscuous IDP that uses the same linear motif to fold into two distinct conformations upon binding to two different partners. By combining ITC experiments with NMR exchange techniques, we have determined both the thermodynamic profiles and interaction kinetics of the two complexes. The unfavorable entropic contribution to binding arising from α-helical folding in the case of the JNK1 complex is compensated by a more favorable binding enthalpy (compared to the p38α complex) arising from the formation of two intra-helical hydrogen bonds. The exchange experiments demonstrate that the three-times higher binding affinity for the JNK1 complex mainly arises from an off-rate contribution. The on-rate for IDP complexes is in many cases dominated by electrostatic interactions (“steering”) through charged re-sidues surrounding the binding pocket [52]. The high sequence conservation and structural homology of JNK1 and p38α (Figures S1–S3) would therefore be in agreement with a similar on-rate for the two complexes. The off-rate presumably is dependent on the number of established interactions in the complex. Comparison of the solved crystal structures does not immediately reveal a difference in the number of established hydrogen bonds between the docking site peptides and JNK1 and p38α. This suggest that the partly helical conformation adopted by MKK4 in complex with JNK1 better complements the narrower JNK1 binding pocket than the extended conformation of MKK4 in the wider p38α groove. We thus propose that the slower off-rate of the JNK1 complex arises from more favorable hydrophobic interactions being established in the complex due to ideal shape complementarity of the two proteins and/or from a rate-limiting helical unfolding step. 

We note that extensive crystallization trials, carried out by us and others [33], have systematically failed for both complexes, highlighting the need for new methods to structurally resolve the binding modes of docking site motifs and IDPs in general. In this context, NMR exchange techniques can be extremely powerful for probing structural transitions through changes in chemical shifts, even for high molecular weight partners [53]. Using these techniques, in combination with other biophysical methods, we therefore now possess the necessary tools to monitor both structure, dynamics, interaction kinetics and thermodynamic profiles of IDP complexes. This will be key to resolving the enigma of affinity versus specificity for IDPs and more generally to understand the ubiquitous role of IDPs in biology.

## Supplementary Materials

The following are available online at https://www.mdpi.com/article/10.3390/biom11081204/s1, Figure S1: Pairwise sequence alignment of JNK1 and p38α, Figure S2: Structural alignment of JNK1 and p38α, Figure S3: Surface charge and hydrophobicity distribution of p38α and JNK1, Figure S4: ^15^N CEST data of MKK4 in its free state, Figure S5: Ramachandran plots showing the distribution of backbone dihedral angles in docking site motifs of JNK1 and p38α.
