# Supplementary material for: Enthalpy–Entropy Compensation in the Promiscuous Interaction of an Intrinsically Disordered Protein with Homologous Protein Partners
Source: Biomolecules. 2021 Aug 13;11(8):1204. doi: 10.3390/biom11081204 (PMC8391806; doi:10.3390/biom11081204)
Supplement: Supplementary file 1 [file biomolecules-11-01204-s001.zip › biomolecules-1317970-supplementary.pdf]

Supplementary Materials

Enthalpy-entropy compensation in the promiscuous interaction of an intrinsically disordered protein with homologous protein partners

Jaka Kragelj[1,†,‡], Thibault Orand[1,†], Elise Delaforge[1], Laura Tengo[1,§], Martin Blackledge[1], Andrés Palencia[2], Malene Ringkjøbing Jensen[1*]

[1]Université Grenoble Alpes, CEA, CNRS, Institut de Biologie Structurale, Grenoble, France
[2]Institute for Advanced Biosciences, Structural Biology of Novel Targets in Human Diseases, INSERM U1209, CNRS UMR5309, Université Grenoble Alpes, Grenoble, France
[†]These authors contributed equally to this work
[‡]Present address: Department of Biophysics, University of Texas Southwestern Medical Center, Dallas, TX 75390, USA
[§]Present address: European Molecular Biology Laboratory, Grenoble, France
*To whom correspondence should be addressed
Dr. Malene Ringkjøbing Jensen
E-mail: malene.ringkjobing-jensen@ibs.fr
Tel: +33 4 57 42 86 68

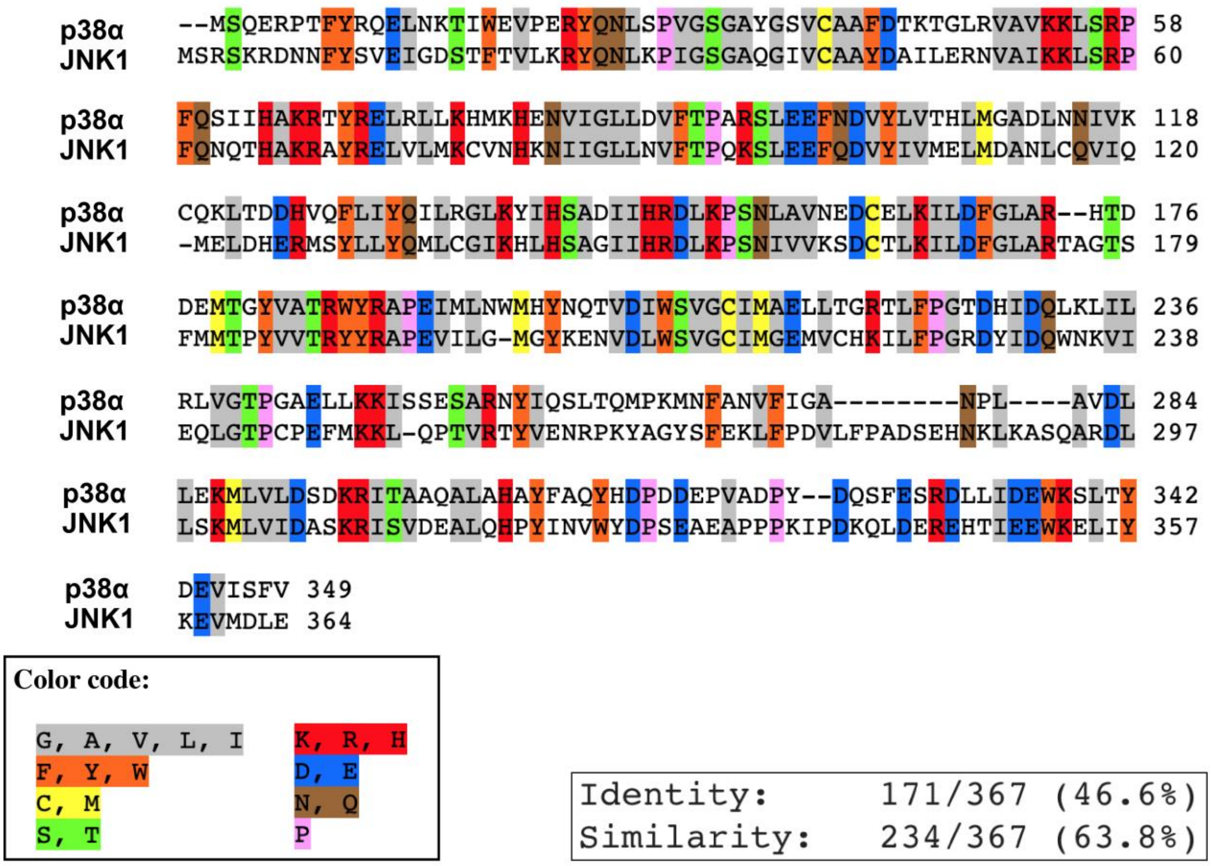

Figure S1. Pairwise sequence alignment of JNK1 and p38α using EMBOSS Needle [1]. Only the portion of the sequences observable in the X-ray structures were used for the alignment.

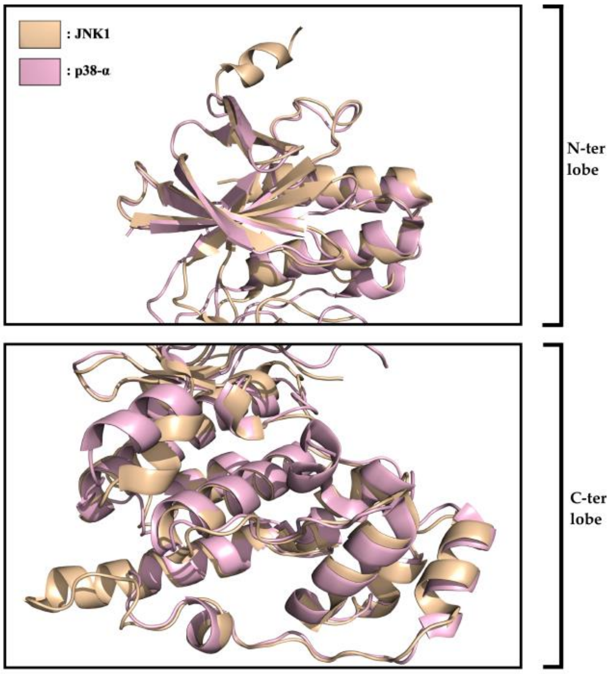

Figure S2. Structural alignment of JNK1 (PDB 2XRW, wheat) and p38α (PDB 1LEW, pink). The N-terminal (upper panel) and C-terminal (lower panel) lobes were aligned separately to avoid any bias from different inter-lobe orientations in the two crystal structures.

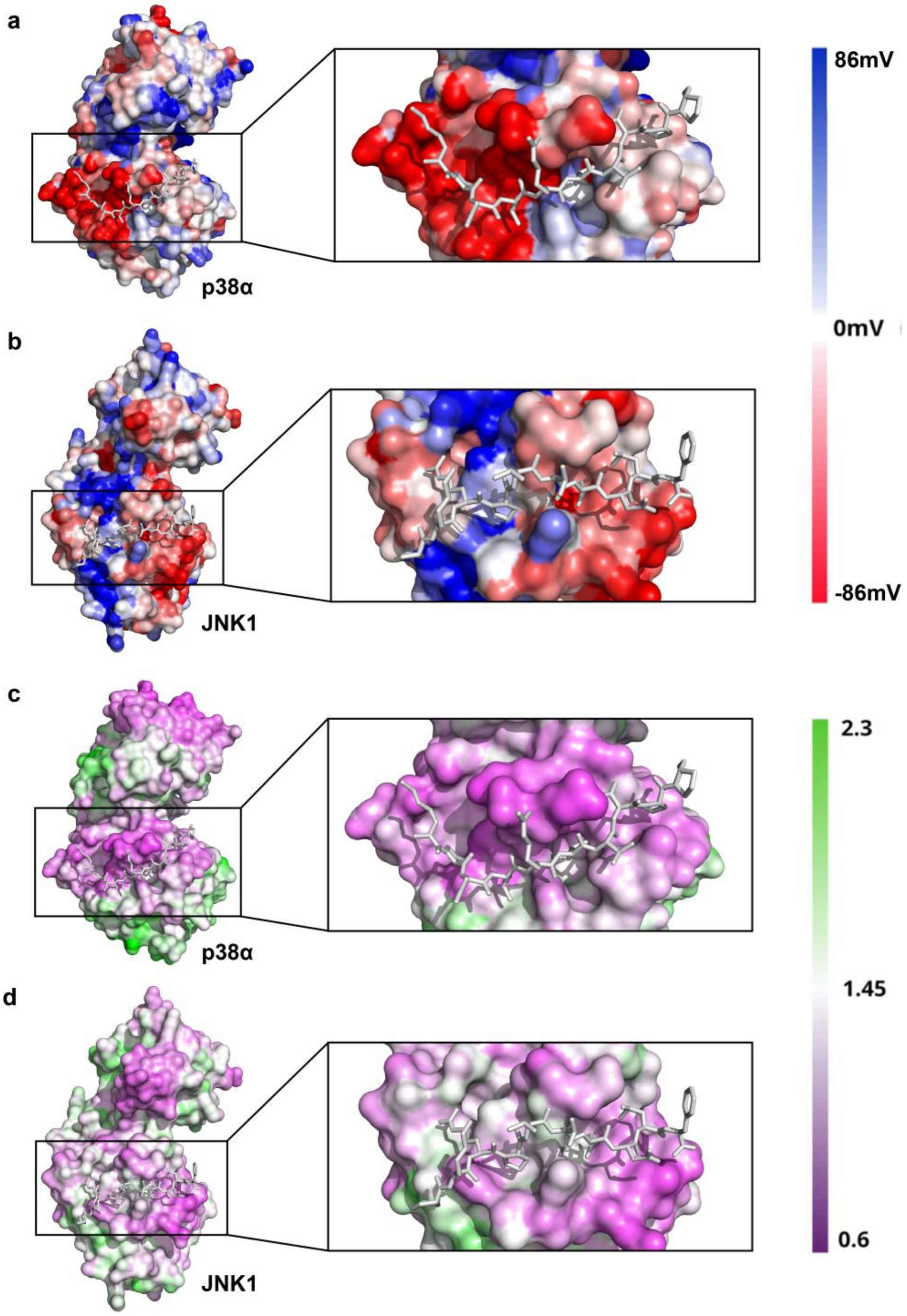

Figure S3. Surface representation of p38α (panel a and c, PDB 1LEW) and JNK1 (panel b and d, PDB 1UKH) showing their charge (panel a and b) and hydrophobicity (panel c and d) distributions. The zooms show a closer view of the docking grooves accommodating the docking site motifs. The charge distribution (in mV) was calculated at pH 6.3, and for the hydrophobicity, the surface is color coded according to the ratio of non-polar versus polar residues. The surface properties were calculated using the webserver Protein-Sol [2].

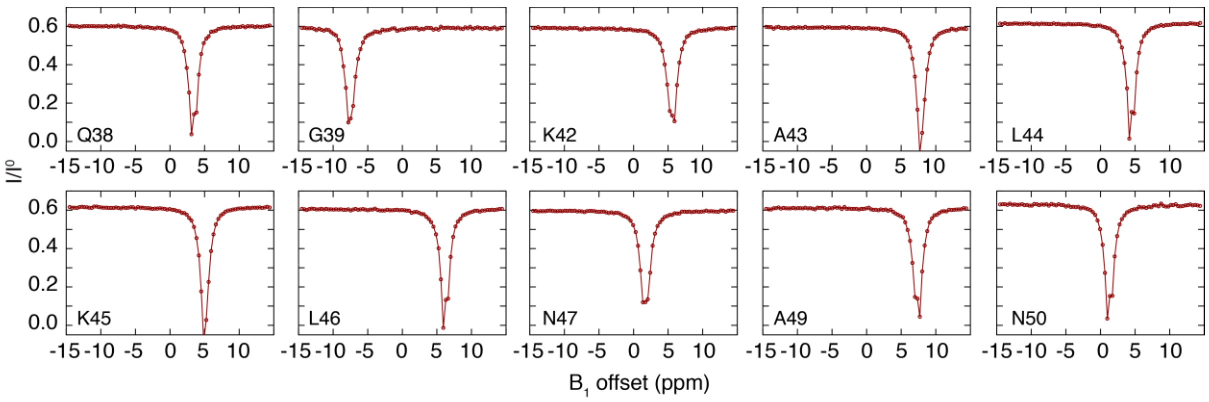

Figure S4. ^{15}N CEST data of MKK4 in its free state. Data are shown for the same residues as in Figure 5a of the main text.

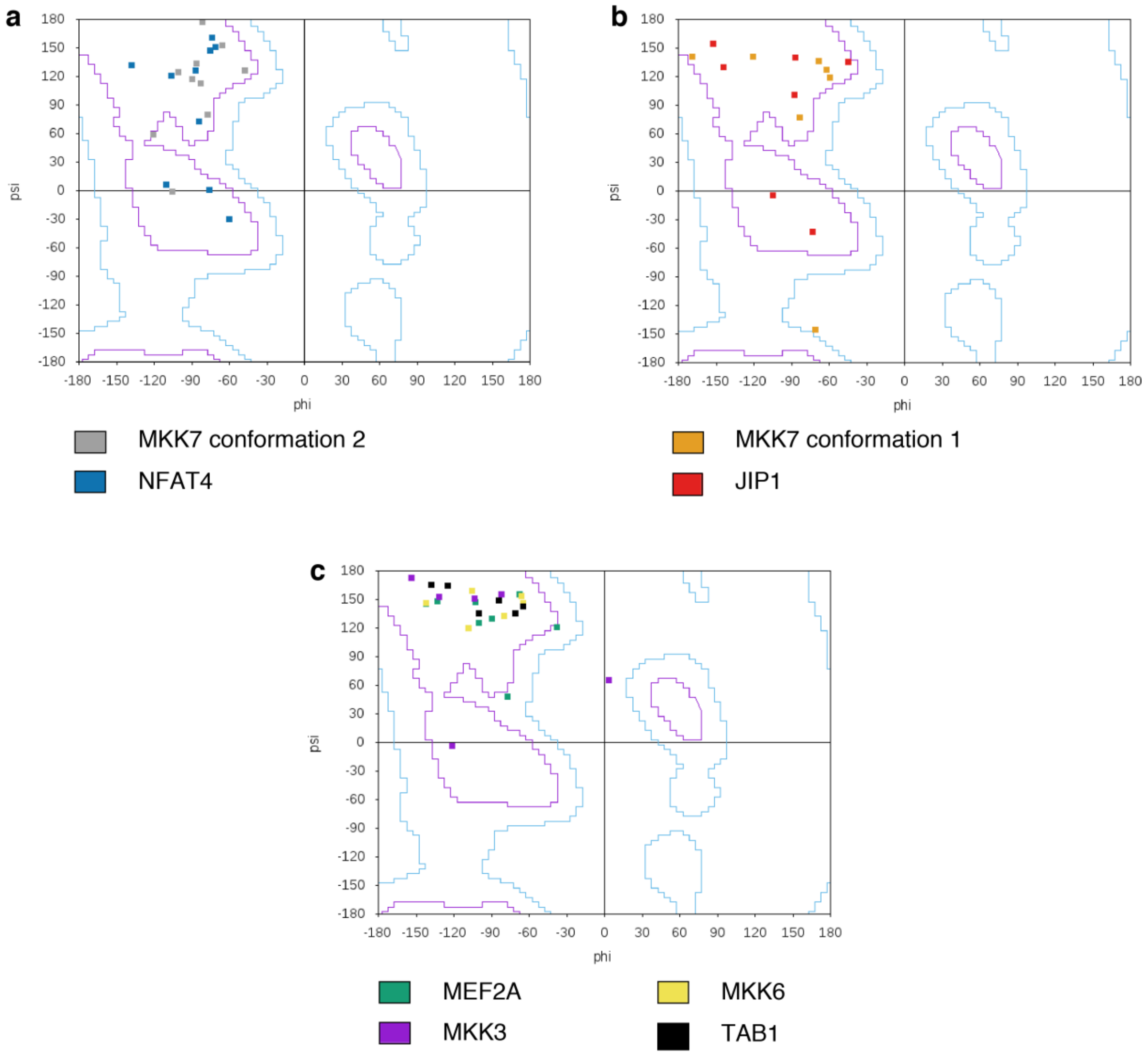

Figure S5. Ramachandran plots showing the distribution of backbone dihedral angles in docking site motifs of JNK1 (**a**-**b**) and p38α (**c**). The structures shown in Figure 6 were used to generate the Ramachandran plots. Most favored regions are indicated by magenta contours, while additional allowed regions are indicated by blue contours.

References

1. Madeira, F.; Park, Y.M.; Lee, J.; Buso, N.; Gur, T.; Madhusoodanan, N.; Basutkar, P.; Tivey, A.R.N.; Potter, S.C.; Finn, R.D.; et al. The EMBL-EBI Search and Sequence Analysis Tools APIs in 2019. *Nucleic. Acids Res.* **2019**, *47*, W636–W641.
2. Hebditch, M.; Warwicker, J. Web-Based Display of Protein Surface and PH-Dependent Properties for Assessing the Developability of Biotherapeutics. *Sci. Rep.* **2019**, *9*, 1969.
